# Description of the Protocols for Randomized Controlled Trials on Cancer Drugs Conducted in Spain (1999–2003)

**DOI:** 10.1371/journal.pone.0079684

**Published:** 2013-11-13

**Authors:** Xavier Bonfill, Mónica Ballesteros, Ignasi Gich, María Antonia Serrano, Fernando García López, Gerard Urrútia

**Affiliations:** 1 Iberoamerican Cochrane Centre, Sant Pau Biomedical Research Institute (IIB Sant Pau), Barcelona, Spain; 2 CIBER Epidemiología y Salud Pública (CIBERESP), Barcelona, Spain; 3 Universitat Autònoma de Barcelona, Barcelona, Spain; 4 PhD Program, Department of Pediatrics, Obstetrics and Gynecology, and Preventive Medicine, Universitat Autònoma de Barcelona, Barcelona, Spain; 5 Agencia Española de Medicamentos y Productos Sanitarios, Madrid, Spain; 6 Nephrology Department, University Hospital Puerta de Hierro, Majadahonda, Madrid, Spain; Davidoff Center, Israel

## Abstract

**Objective:**

To describe the characteristics of randomized controlled clinical trials (RCT) on cancer drugs conducted in Spain between 1999 and 2003 based on their protocols.

**Methods:**

We conducted an observational retrospective cohort study to identify the protocols of RCTs on cancer drugs authorized by the Agencia Española del Medicamento y Productos Sanitarios (AEMPS) (Spanish Agency for Medicines and Medical Devices) during 1999-2003. A descriptive analysis was completed and the association between variables based on the study setting and sponsorship were assessed.

**Results:**

We identified a total of 303 protocols, which included 176,835 potentially eligible patients. Three-quarter of the studies were internationally-based, 61.7% were phase III, and 76.2% were sponsored by pharmaceutical companies. The most frequently assessed outcomes were response rate (24.7%), overall survival (20.7%), and progression-free survival (14.5%). Of all protocols, 10.6% intended to include more than 1000 patients (mean: 2442, SD: 2724). Compared with their national counterparts, internationally-based studies were significantly larger (p<0.001) and were more likely to implement centralized randomization (p<0.001), blinding of the intervention (p<0.001), and survival as primary outcome (p<0.001). Additionally, most internationally-based studies were sponsored by pharmaceutical companies (p<0.01). In a high percentage of protocols, the available information was not explicit enough to assess the validity of each trial. Compared to other European countries, the proportion of Spanish cancer drugs protocols registered at www.clinicaltrials.gov (7%) was lower.

**Conclusion:**

RCTs on cancer drugs conducted in Spain between 1999 and 2003 were more likely to be promoted by pharmaceutical companies rather than by non-profit national groups. The former were more often part of international studies, which generally had better methodological quality than national ones. There are some worldwide on-going initiatives that aim to increase the transparency and quality of future research.

## Introduction

Randomized controlled clinical trials (RCTs) are the best available study design for assessing the effectiveness of health care interventions [1]. Moreover, conducting RCTs is an ineludible prerequisite of regulatory agencies for authorizing the commercialization of new treatments or for approving new indications or uses for a given drug [2]. It is important that the main characteristics of each trial are made available before the study is conducted. This measure facilitates scrutiny of design by peers and other experts, encourages publication, and promotes full outcome reporting [3]. In order to achieve this goal, some institutions such as the World Health Organization [4], The Cochrane Collaboration [5], and the International Committee of Medical Journal Editors recommend prospective registration of all RCTs in publicly accessible registries [6].

Every RCT must be conducted in accordance with a corresponding protocol, which must specify study rationale, methodology, logistics, and ethical considerations, among other aspects. Therefore, protocols provide the blueprint for planning all stages of a study, from participant recruitment to results dissemination [7,8]. In most countries, protocols and other complementary documents must be approved by an ethic research committee before clinical trials can be conducted. 

In Spain, all RCTs must receive authorization from the Agencia Española del Medicamento y Productos Sanitarios (AEMPS) (Spanish Agency for Medicines and Medical Devices) and be registered in the AEMPS clinical trial registry. This registry is a pioneering initiative worldwide [9-12] that allows assessment of different aspects related to the quality of RCTs. 

The objective of our study was to analyze different aspects of RCTs on cancer drugs authorized in Spain. In this article, we will describe our main findings based on the information available in the protocols from the period between 1999 and 2003. We also identified all publications derived from these studies by carrying out electronic searches in bibliographic databases and by contacting sponsors or researchers. Details of this project will be provided in forthcoming papers that focus on dissemination bias and other issues related to methodological quality.

## Materials and Methods

We conducted an observational retrospective cohort study, which included all protocols for RCTs on cancer drugs authorized by the AEMPS between 1999 and 2003. This period was selected to allow enough time for potential study completion and publication of results. Only RCTs with a control group and adequate randomization, regardless of study phase (II to IV), were included. 

We identified RCTs on cancer drugs from the AEMPS database by assessing all titles registered within the period of interest. We then manually assessed, if available, their corresponding protocols and extracted information on design, participants, interventions, and outcome measures, among other variables. These data were reviewed, edited, and subsequently entered into a database. Additionally, we identified RCTs that were registered in ClinicalTrials.gov and determined if their protocols included the 20 items required by the World Health Organization [13].

We used measures of central tendency (media and median) and dispersion (standard deviation and range) in the descriptive analysis of quantitative variables, as well as proportions for qualitative variables. The Pearson's chi-squared test for categorical variables χ^2^ was used to analyze possible associations among variables. Statistical significance was considered for p-values ≤0.05.

Since this research project did not involve patients or the use of clinical data, we did not request approval form our institution´s ethics committee, which is in agreement with Spanish Legislation on biomedical research (Ley 14/2007). 

## Results

We identified 303 protocols for RCTs that had been approved during the period of interest. In total, these RCTs planned to include 176,835 cancer patients. The most frequently studied tumor locations were: breast in 70 studies (23.1%), lung in 50 (16.5%), colorectal in 20 (6.6%), ovary in 19 (6.3%), prostate in 18 (5.9%), lymphoma in 16 (5.3%), head and neck in 12 (4.0%), and other locations in 79 (26.0%). Nineteen studies evaluated the treatment of cancer-related symptoms (6.3%), without specifically focusing on tumor location. The main characteristics of included RCTs are shown in table 1.

**Table 1:** Differences by setting (national versus international) of the main characteristics of the protocols authorized by the AEMPS between 1999 and 2003.

		**Setting (%)**			
**Variable**		**National (67)**	**International (236)**	**Total**	P*
**Year of authorization**					
1999		13 (19.4)	44 (18.6)	57	0.90
2000		14 (20.8)	54 (22.8)	68	
2001		15 (22.3)	48 (20.3)	63	
2002		12 (17.9)	34 (14.4)	46	
2003		13 (19.4)	56 (23.7)	69	
**Type of hypothesis**					
Non-inferiority		13 (19.4)	39 (16.5)	52	0.58 NS
Superiority		54 (80.5)	197 (83.4)	251	
**Sample size**					
0-100		19 (28.3)	28 (11.8)	47	0.00
101-500		37 (55.2)	116 (49.1)	153	
501-1000		5 (7.4)	62 (26.2)	67	
More than 1001		5 (7.4)	27 (11.4)	32	
No data		1 (1.4)	3 (1.2)	4	
**Type of sponsor**					
Academic		37 (55.2)	35 (14.8)	72	0.00
Pharmaceutical industry		30 (44.7)	201 (85.1)	231	
**RCT phase**					
Phase II		26 (38.8)	67 (28.3)	93	0.00
Phase III		29 (43.2)	158 (66.9)	187	
Phase IV		12 (17.9)	11 (4.6)	23	
**Groups of comparison**					
Different medicinal products		35 (52.2)	141 (59.7)	176	0.36
Different combinations of a single drug		21 (31.3)	51 (21.6)	72	
Medicinal products versus observation or placebo		11 (16.4)	42 (17.7)	53	
Other		0 (0)	2 (0.8)	2	
**Type of randomization**					
Centralized		45 (67.1)	201 (85.1)	246	0.00
Non-centralized		6 (8.9)	8 (3.3)	14	
No data		16 (23.8)	27 (11.4)	43	
**Type of masking**					
Open-label		56 (83.5)	149 (63.1)	205	0.00
Blind		11 (16.4)	87 (36.8)	98	
**Primary outcome**					
Response rate		29 (43.9)	46 (19.4)	75	0.00
Overall survival		3 (4.5)	50 (21.1)	53	
Progression-free survival		3 (4.5)	33 (13.9)	36	
Disease-free survival		8 (12.1)	19 (8.0)	27	
Time to progression		3 (4.5)	11 (4.6)	14	
Time to treatment failure		2 (3.0)	10 (4.2)	12	
Symptom control		2 (3.0)	7 (3.0)	9	
Safety/toxicity		5 (7.6)	3 (1.3)	8	
Relative survival		1 (1.5)	6 (2.5)	7	
Quality of life		1 (1.5)	3 (1.3)	4	
Other		7 (10.6)	45 (19.0)	52	
No data		2 (3.0)	4 (1.7)	6	

NS: Non-significant

* Significance level

The mean number of RCTs protocols authorized per year was 60.6 (range 46-69), with no specific trend during the studied period. There were more phase III studies (61.7%) than phase II (30.6%) or phase IV (7.5%) studies. Of all protocols, 67 (22.1%) corresponded to exclusively national studies (with a mean of 10.6 centers expected to participate) compared to 236 (77.8%) international studies.

Most RCTs (76.2%) were sponsored by pharmaceutical companies, while the remaining 23.8% had non-commercial sponsors. Commercial sponsorship was more significant among international studies (85.1%) than among national studies (44.7%) (p=0.00). Investigational medicinal products requiring product under clinical research qualification, which are novel medicinal products that have not been authorized in Spain and containing an active substance not included in any of the authorized medicinal products in Spain, were the most common studies (40.5%). There were also RCTs on medicinal products with a marketing authorization used either in conditions different from those previously authorized (dose, route, etc.) (18.5%), a new indication (14.5%), or in the same conditions of use as authorized (14.1%). The rest of the RCTs intended to assess new indications, dosage, and route of administration (<1%). In 11.5% of protocols, the authorization status of the investigational medicinal product was not stated. Randomization was centralized in 81.2% of the studies, while no detailed information on this matter was available for 14.2%. Only 32.3% of the studies had some masking of the intervention (single or double-blind) whereas 67.7% had an open design. RCTs with a superiority hypothesis (82.8%) were more common than those with a non-inferiority hypothesis (17.2%). 

Overall, the analyzed protocols referred to 641 treatment groups, including studies with 2 comparative groups (83.2%), 3 comparative groups (10.8%), and between 4 and 6 comparative groups (6.0%). Regarding type of comparison, 176 (58.1%) RCTs compared different drugs or therapeutic regimens (A vs B; A+B vs C+D), 72 (23.8%) compared different combinations of a single drug (A+B vs A), and 53 (18.0%) compared a single drug or a combination of drugs against observation or placebo. One study compared different initiation times of a single treatment.

In total, 611 investigational medicinal products were used, corresponding to 166 different active substances, both antineoplastic and non-antineoplastic. The most frequently evaluated antineoplastic agents were cisplatin (5.7%), docetaxel (5.7%), 5-fluorouracil (4.2%), carboplatin (4.2%), and paclitaxel (4.2%), as well as regimens with multiple medications (in 178 cases). The most frequently studied non-antineoplastic agents were dexamethasone (1.4%), epoetin alpha (1.1%), prednisone (0.9%), and aprepitan (0.6%). 

The most frequently assessed primary outcomes were response rate (24.8%), overall survival (20.7%), progression-free survival (14.2%), incidence of cancer (10.2%), disease-free survival (8.9%), time-to-treatment failure (6.6%), control of symptoms (3.9%), safety/toxicity (3.0%), quality of life (1.3%), and other outcomes (3.9%). The primary outcome was unclear in 3.6% of the protocols.

The mean number of expected patients per trial according to the protocols was 591 (SD: 1169), ranging between 15 and 15,000 (median: 328). Up to 500 patients were to be included in 66.8% of the studies, between 501 and 1000 in 22.1%, and more than 1001 in 10.6% (especially breast cancer studies). Sample size was not justified in 4 protocols.

Compared to national studies, international studies were more likely to include larger sample sizes, have pharmaceutical companies as sponsors, be phase III studies , implement central randomization, mask the intervention, and use survival as the primary outcome (see Table 1 for the comparison between national and internationals RCTs).

Of the 246 RCTs authorized in the period between 2000 and 2003, 35 (7%) were registered at ClinicalTrials.gov. All items recommended by the WHO were included in 70% of the protocols. 

## Discussion

Cancer is the clinical area in which the largest number of RCTs on medicinal products are conducted worldwide [14]. In Spain, the number of trials in this area accounts for about a third of the total RCTs [15] and for about one fourth of those registered at ClinicalTrials.gov [16]. Therefore, it is of great interest to describe in detail the characteristics of RCTs developed in the field of Oncology. This, in turn, would foster an analysis of some measures that might improve their quality and clinical relevance. 

RCT protocols allow ethical committees and regulatory authorities to assess the scientific, ethical, and administrative parameters of a study. They are the basis for the information provided to participants and guide researchers and sponsors as trials are conducted. Additionally, protocol amendments allow tracking relevant changes introduced as RCTs are underway [7]. 

This paper describes the original protocols of RCTs in Oncology identified in the prospective registry of the AEMPS submitted and authorized in Spain between 1999 and 2003. Data contained in the RCTs protocols are more complete than those found in existing international registries. However, only a few studies using data of RCT protocols have been published to date, and they focus mostly on aspects that need to be improved in order to increase the relevance and applicability of protocols [15,17]. 

A relevant finding of our study is that half of the authorized RCTs studied new molecules or new indications of previously approved medications. This pattern has increased over time in different countries [15,18], which denotes the high degree of innovation and commercial interest in this important clinical area. 

Approximately three-quarters (77.8%) of the RCTs identified in this study were internationally-based, most of which were sponsored by pharmaceutical companies. However, it is not always possible to detect non-explicit, commercial, indirect sponsorships [19]. We plan to investigate this issue in the near future. As expected, international RCTs had a larger sample size (a third included more than 500 participants, compared with 15.0% in national based studies). 

Phase III RCTs were more likely to be internationally-based than nationally-based. This may be explained by a greater proportion of studies sponsored by pharmaceutical companies, which are conducted for regulatory purposes of new investigational medicinal products, and that require a more demanding methodological approach. On the other hand, the relatively higher proportion of phase IV trials among national studies possibly reflects the different nature of RCTs promoted by cooperative groups. These RCTs are more centered on the comparative effectiveness of treatments already authorized and/or on exploring aspects of clinical interest that do not necessarily have a commercial purpose. This interpretation is reinforced by the fact that international trials mostly tested superiority hypotheses for new investigational medicinal products. 

Some authors report that novel treatments are not always better than the conventional ones [20-22] and that RCTs sponsored by the pharmaceutical industry are more likely to report treatment success when compared with an already-existing intervention [23]. In addition, the clinical relevance of the observed effect is often limited [24]. We are currently conducting a study to determine what percentage of the identified trials evaluate a new intervention report treatment success, as well as the relationship, if any, with the study sponsor. This would be the object of a forthcoming paper. Furthermore, future studies should explore the impact that new treatments have on the clinical management of cancer in the clinical setting in terms of benefits for patients. 

Regarding quality of RCTs, international RCTs with centralized randomization and blinding were associated with higher standards in their design, compared to national RCTs. In contrast, academic RCTs with no declared commercial interest or support seemed to be conducted with lower methodological rigor, possibly due to financial or logistical constraints. The lack of details found in the methods section of the protocols of these RCTs suggests that investigators without commercial support were not as familiar with the current legislation on designing RCTs as their counterparts in the pharmaceutical companies. In fact, the variability in protocol content and quality that we encountered limited our analysis

Only 7.0% of the RCTs identified in this study were registered at ClinicalTrials.gov, which is lower than what has been seen in other European countries. The fact that during this period of time prospective registry of trials in ClinicalTrials.gov was mandatory only for trials conducted in the US may explain that low percentage. In addition, it was not until the year 2005 that the International Committee of Medical Journals Editors requested prospective registry of trials a requirement for publication [6]. Much less likely, however, is that some protocols were registered in public databases that were not explored in this study. In addition, the annual mean of Spanish cancer RCTs registered at ClinicalTrials.gov in the period under study (33.2) is much lower than those of countries such as Germany (53.0), France (56.5), or Canada (117.5), which might indicate a smaller research activity in Spain in the area of cancer or a lower registration rate of protocols in that platform.

Our study is pioneer in the sense that it exhaustively describes the main characteristics of RCTs on cancer conducted in Spain. However, it has some potential limitations. First, the selection criteria resulted in the exclusion of phase I and II clinical trials with no control group or random allocation, which is a common design in the field of Oncology (e.g. dose-finding studies). This might underestimate the real number of clinical trials on cancer authorized in Spain. However, we focused on phase III and IV RCTs because they have a higher potential clinic impact. In addition, we were only able to study trials on medicinal products because during the period of time analyzed, the AEMPS only had jurisprudence over this type of trials.

Additionally, we only had full access to the initially authorized protocols but not to amendments, final reports, etc. This did not allow us to detect possible changes in the protocols or other contingencies that may have occurred as the studies were conducted. It also prevented us from crosschecking adherence to the protocol (e.g. the number of included patients, or the participating centers). 

Our work reinforces the usefulness of making the registry of all authorized trials compulsory in publicly accessible registries [25]. The AllTrials campaign [26], launched in 2012 with broad international support, calls for the registration of all RCTs in public registries, which in turn may guarantee free access to all relevant information of the study and results, including individual patient data rather than just summaries or aggregate data.

 Furthermore, the new European Union regulation on clinical trials currently under discussion specifies the key items that should be included in a RCT protocol based on the ICH guidelines (19, 20, 21). This regulation advocates for free access to relevant information of protocols as well as to all pertinent modifications and amendments. However, some authors have already raised concerns about potential limitations of the new regulation [27]. In addition, the recently published initiative SPIRIT 2013 Statement provides a more detailed guideline specifying the expected content of a RCT protocol [28] that eventually will help improve the quality of RCT protocols by non-profit sponsors.

In conclusion, access to the information contained in trial protocols allow the description and analysis of research conducted in a particular field. In the case of Spanish oncological RCTs, they were much more likely promoted by pharmaceutical companies than by non-profit national groups. The former were often part of international studies, which in general had better methodological quality than the national ones. There are some worldwide on-going initiatives that aim to make all the process and data related to conducting clinical trials more explicit and accessible, which should increase the transparency and quality of future research.
